# Unintended Consequences of Management Actions in Salt Pond Restoration: Cascading Effects in Trophic Interactions

**DOI:** 10.1371/journal.pone.0119345

**Published:** 2015-06-01

**Authors:** John Y. Takekawa, Joshua T. Ackerman, L. Arriana Brand, Tanya R. Graham, Collin A. Eagles-Smith, Mark P. Herzog, Brent R. Topping, Gregory G. Shellenbarger, James S. Kuwabara, Eric Mruz, Sara L. Piotter, Nicole D. Athearn

**Affiliations:** 1 U.S. Geological Survey, Western Ecological Research Center, San Francisco Bay Estuary Field Station, 505 Azuar Drive, Vallejo, CA 94592, United States of America; 2 U.S. Geological Survey, Western Ecological Research Center, Dixon Field Station, 800 Business Park Drive, Suite D, Dixon, CA 95620, United States of America; 3 U.S. Geological Survey, Forest and Rangeland Ecosystem Science Center, 3200 SW Jefferson Way, Corvallis, OR, 97331, United States of America; 4 U.S. Geological Survey, National Research Program, 345 Middlefield Road, Mail Stop 466, Menlo Park, CA 94025, United States of America; 5 U.S. Geological Survey, California Water Science Center, Placer Hall, 6000 J Street, Sacramento, CA 95819, United States of America; 6 Don Edwards San Francisco Bay National Wildlife Refuge, U.S. Fish and Wildlife Service, Newark, CA 94560, United States of America; Texas A&M University at Galveston, UNITED STATES

## Abstract

Salt evaporation ponds have played an important role as habitat for migratory waterbirds across the world, however, efforts to restore and manage these habitats to maximize their conservation value has proven to be challenging. For example, salinity reduction has been a goal for restoring and managing former salt evaporation ponds to support waterbirds in the South Bay Salt Pond Restoration Project in San Francisco Bay, California, USA. Here, we describe a case study of unexpected consequences of a low-dissolved oxygen (DO) event on trophic interactions in a salt pond system following management actions to reduce salinity concentrations. We document the ramifications of an anoxic event in water quality including salinity, DO, and temperature, and in the response of the biota including prey fish biomass, numerical response by California Gulls (*Larus californicus*), and chick survival of Forster's Tern (*Sterna forsteri*). Management actions intended to protect receiving waters resulted in decreased DO concentrations that collapsed to zero for ≥ 4 consecutive days, resulting in an extensive fish kill. DO depletion likely resulted from an algal bloom that arose following transition of the pond system from high to low salinity as respiration and decomposition outpaced photosynthetic production. We measured a ≥ 6-fold increase in biomass of fish dropped on the levee by foraging avian predators compared with weeks prior to and following the low-DO event. California Gulls rapidly responded to the availability of aerobically-stressed and vulnerable fish and increased in abundance by two orders of magnitude. Mark-recapture analysis of 254 Forster's Tern chicks indicated that their survival declined substantially following the increase in gull abundance. Thus, management actions to reduce salinity concentrations resulted in cascading effects in trophic interactions that serves as a cautionary tale illustrating the importance of understanding the interaction of water quality and trophic structure when managing restoration of salt ponds.

## Introduction

Throughout the world, wetlands support a disproportionate number of endemic and endangered vertebrate species including fish and waterbirds which are threatened by water quality degradation [1–3]. Many of these important wetland habitats are experiencing increased salinization from altered freshwater flows, reduced infiltration, seawater encroachment, and commercial salt production [4–6]. In addition, elevated salinity in ground or surface waters affects nutrient and toxicant bioavailability [7] as well as community composition and associated aquatic food webs [5, 8].

Aquatic plants and animals often tolerate a relatively narrow salinity range, and salinity reduction can be an important restoration or management tool to enhance prey populations for target predators [6]. Restoration of freshwater inputs increased demersal fish populations that served as a food base for piscivorous birds in brackish to oligohaline wetlands of the Florida Everglades [4]. In China’s Yellow River Delta, hydrologic reconnection between the river and floodplain reduced soil salinity and resulted in rapid colonization of aquatic vegetation and subsequent increases in avian species richness that were likely related to the increased food base [9]. Whereas salinity reduction can benefit predator species, hypoxia associated with the rapid increase in abundance of species may create unintended problems that can hamper wetland restoration efforts [2, 3, 6].

Warmer lentic water bodies with high nutrients and long residence times are subject to eutrophication that depletes dissolved oxygen (DO) and consequently alters predator-prey interactions [1, 3, 10]. Fish kills in hypoxic coastal wetlands disrupt the aquatic food chain by altering the composition and biomass of prey assemblages and causing dietary shifts by predators [2, 10, 11]. Suboxic conditions in salt marsh pools from diurnal photosynthetic cycles can stimulate aquatic surface respiration in fishes that increases their vulnerability to avian predators [12, 13]. Additionally, prey responses to depleting DO can affect predator feeding strategies, such as the timing, method, or location of foraging by piscivorous birds that affects their success rates [12, 13, 14]. Rapid shifts in abiotic conditions, such as DO depletion, can alter species reactions and trophic interactions in surprising and unexpected ways, particularly at upper trophic levels that may be less able to respond [10, 15, 16].

Recently, > 6100 ha of former commercial salt evaporation ponds were purchased in San Francisco Bay for re-establishment of tidal marshes or for management favoring waterbirds in one of the largest restoration projects in western North America. Salinity reduction increases diversity and abundance of benthic invertebrates and fish with apparent benefits for shorebirds, diving ducks, and piscivorous birds [5, 17–20]. The goal of the restoration program has focused on providing a mosaic of habitat types including management of 10–50% of the area as open-water ponds to support foraging for hundreds of thousands of waterbirds representing > 70 species that use the area to nest, migrate, or over-winter [21]. Initial management actions are targeting reduction of salinities and increasing invertebrate and fish prey resources [5, 17, 20]. In this study, we use our restoration monitoring datasets which include waterbird surveys, nest monitoring, and water quality measurements to describe and explain the unexpected trophic consequences of salinity reduction management actions in the salt pond system. For the first time, we provide detailed information that explains how such an event can occur, and we discuss our results in context of the general importance of reducing risks of hypoxia for managing wetlands.

## Methods

### Study Area

**Fig 1:**
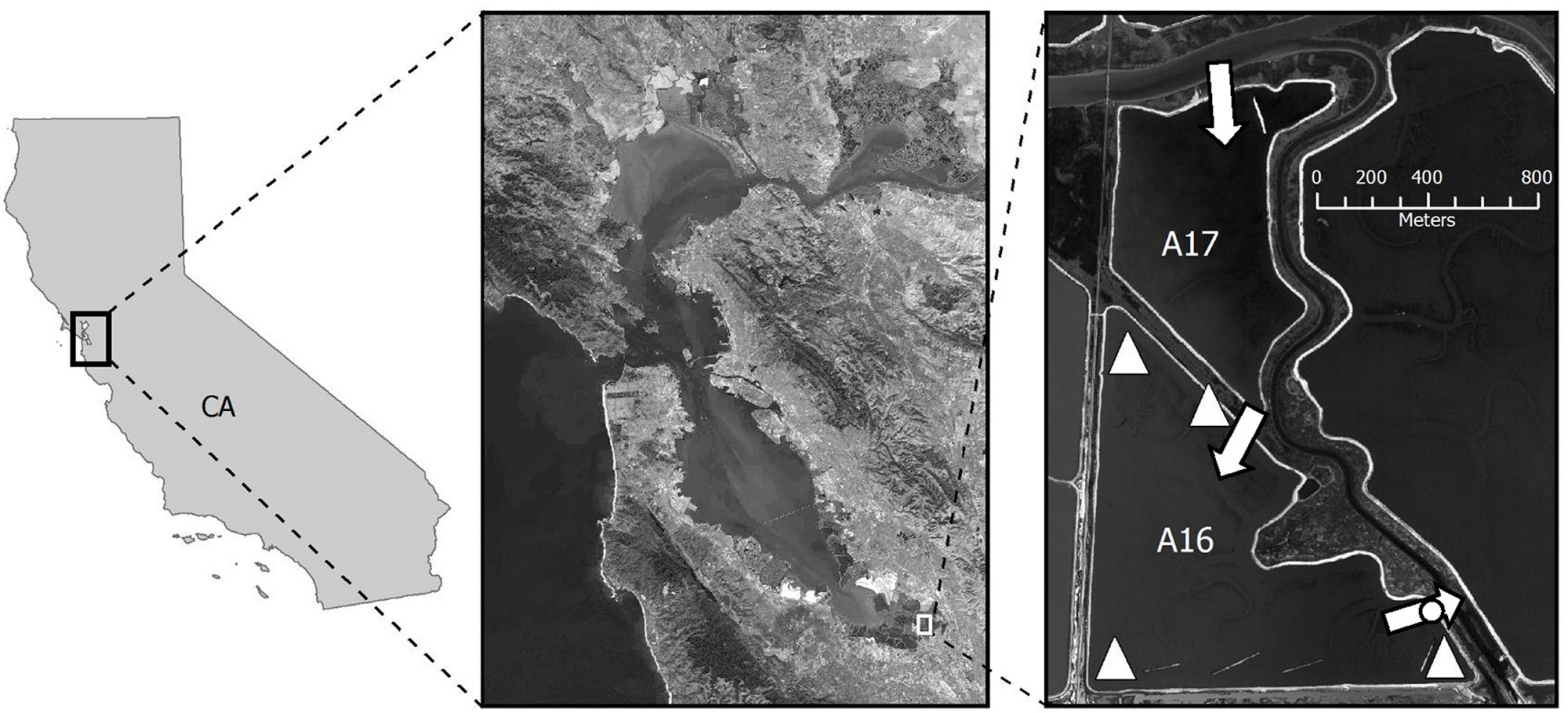
Study location in South San Francisco Bay, California, Alviso Pond A17 (north) and Pond A16 (south). Arrows indicate the dominant direction of flow from Coyote Creek in the north through the inflow gate to Pond A17, through the uncontrolled internal levee channel into Pond A16, and out the A16 discharge gate in the southeast corner into Alviso Slough. The circle indicates datasonde location; triangles indicate minisonde sampling locations. Forster’s Tern nesting colonies occurred on the four linear islands in the southern part of pond A16. Imagery provided by Landsat 8 and 2005 National Agricultural Imagery Program (NAIP).

All work was conducted on Refuge land under a Special Use Permit issued by Don Edwards San Francisco Bay National Wildlife Refuge (SFBNWR). Methods were reviewed and approved by the Animal Care and Use Committee (ACUC) of the Western Ecological Research Center (WERC). The study area was located within the South Bay Salt Pond Restoration Project in San Francisco Bay, California, USA (Fig 1). Twenty-five former salt ponds within the Alviso pond system were acquired in 2003 by the U.S. Fish and Wildlife Service (USFWS) as part of the Don Edwards SFBNWR. Prior to land transfer, commercial ponds were managed for salt extraction wherein bay water was moved through sequential ponds to increase salt concentration by solar evaporation [22]. From summer through late fall 2003–2005, water control structures were installed in 14 ponds to control circulation and reduce salinities as part of the Initial Stewardship Plan for the restoration. With the change in pond management, salinity reduction was followed by increased diversity and abundance of fish [17] and piscivorous and opportunistic birds such as California Gulls [23].

Our study focused on Ponds A16 (101 ha) and A17 (53 ha) located on the eastern side of the Alviso complex (Fig 1). The intake to Pond A17 and the discharge gate from Pond A16 on this two-pond system were the primary portals for water exchange with the adjacent pond and slough. Both gates contained weir boards to regulate flow volume and flap gates that could be set to unidirectional or two-way flow, and exchange between ponds occurred through an open channel in the dividing levee. Management actions to circulate water in the pond began at the end of April 2005.

### Water Quality

As part of the restoration monitoring program, we measured water temperature, salinity, pH, and DO monthly in both ponds during the study period from August 2003 through December 2005 with nearly-continuous readings in Pond A16 from July 2005 through August 2005. We used Hydrolab sondes (Hach Company, Loveland, CO), equipped with Clark cell DO sensors (mg L^-1^), thermometers (°C), pH sensors, and specific conductivity sensors (milliSiemans cm^-1^ converted to the 1978 Practical Salinity Unit, PSU). We took instantaneous measurements 10-cm below the surface from four points around Pond A16 using minisondes (Fig 1) within one week of bird surveys. We also used two Hydrolab datasondes to automatically record water quality data at 15-minute intervals. Datasondes were installed inside a weir box and within Pond A16 at the discharge location (Fig 1); each was secured within a PVC frame that allowed free circulation around the sensors.

We downloaded data from datasondes weekly and cleaned and calibrated specific conductivity, pH, and DO sensors semi-monthly [24]. During downloads we checked battery voltage and deployed a calibrated minisonde side-by-side with the datasonde to validate readings and identify maintenance requirements. We used a copper mesh sock held in place by a nylon stocking to minimize fouling. We screened data with rigorous quality control measures [24] and deleted data if sensors were fouled. We applied a two-point calibration drift correction factor when parameters exceeded acceptable drift limits: ± 3% of the measured value for specific conductance, ± 0.3 mg L^-1^ for DO, and ± 0.2 units for pH.

Information on ambient conditions and weather were obtained from external sources. Temperature, salinity, and DO of ambient surface water conditions in South San Francisco Bay were obtained from U.S. Geological Survey Station 36 (http://sfbay.wr.usgs.gov/access/wqdata/) located 11 km northwest of the study site. Daily-averaged photosynthetically active radiation (PAR; mol quanta m^-2^ s^-1^), wind speed (m s^-1^), and maximum air temperature (°C) were obtained from California Irrigation Management Information Systems Station 171 (www.cimis.water.ca.gov) located 21 km northwest of the study site.

### Bird Populations

To ensure complete coverage of the study area, observers used spotting scopes at several locations around the A16 pond levee, and recorded California Gull abundance on a monthly interval at high tide (≥ 1.2 m or 4 ft MHHW). When gull abundance increased rapidly, our standard protocol was supplemented by photographic surveys in order to ensure an accurate count. On 10 August 2005, we took photographs of Pond A16 and tallied a minimum count of gulls present during the hypoxic conditions that co-occurred with the increased number of gulls (henceforth, gull response) that began on 3 August 2005.

From 16 June 2005 through 17 August 2005, we visited Forster’s Tern nests weekly at the four southern islands that comprised the nesting colony at Pond A16 (Fig 1). This monitoring period, designed to monitor terns throughout the full breeding season at Pond A16, also completely encompassed the hypoxic event during the nesting season. During this period we entered the nesting colony 9 times (each visit separated by 6–8 days) to capture and band newly hatched chicks as well as recapture previously banded chicks and record any observed mortalities. A U.S. Geological Survey stainless steel leg band was placed on the right tarsus of each newly captured chick. For each chick (new and recaptured), we measured mass (± 1 g) with Pesola© spring scales, exposed culmen (± 0.1 mm) and tarsometatarsus (± 0.1 mm) lengths with digital calipers, and flattened wing length (± 1 mm) with a wing board [25]. During capture events, we held birds in shaded poultry cages (model 5KTC, Murray McMurray Hatchery, Webster City, Iowa) and returned them to their capture site.

We estimated chick age by subtracting date of recapture from hatching date if observed. Otherwise we estimated chick age at the first capture date using a structural size model derived from morphometric data of chicks with known hatching dates in San Francisco Bay [26]. Age on subsequent capture dates was calculated based on estimated age at first capture and the difference between the recapture date and initial capture date.

We used Cormack-Jolly-Seber mark recapture models [27] implemented in Program Mark using the R package RMark [28, 29, 30] to estimate daily survival of Forster’s Tern chicks and to assess whether the gull response affected tern chick survivorship. We developed capture histories and used mark-recapture models that accounted for varying interval length between island visits. We examined the influence of three covariates on chick survival: chick age, calendar date, and gull response (before or after 3 August 2005). We also considered the influence of three covariates on encounter probability (probability of chick detection and capture): chick age, calendar date, and a categorical variable to denote whether a chick had fledged after 28 days and was thus unavailable for capture [19]. This yielded a total of 28 candidate models, representing all combinations of the seven survival models (all covariates with additive effects) and four encounter probability models (all covariates with additive effects plus the categorical factor for fledging age always included).

We evaluated models using Akaike’s Information Criterion adjusted for over-dispersion (using median c-hat goodness of fit approach implemented within Program MARK [28]) and small sample size (QAIC_*c*_; $\hat{c}$ = 1.6; [31]). We used Akaike weights (w_*i*_), to assess cumulative evidence in support of each model or variable. In addition, we used evidence ratios to compare the likelihood of two different candidate models [31]. We used the total summed weight for models that incorporated the gull response as a measure of relative variable importance of gull response. We considered values closer to the maximum possible summed weight of 1.0 more important [31]. Finally, survival estimates presented are model-averaged estimates across the full set of candidate models.

### Prey Fish

As an index of prey availability, during weekly Forster’s Tern nest visits, we thoroughly searched nesting colonies for unconsumed fish returned to colonies and deposited by avian predators (hereafter called “dropped fish”). These fish, dropped by parents during nest provision trips to feed their chicks, rapidly desiccate and become unavailable to chicks as forage, but still represent the prey availability to Forster’s Terns and other avian piscivores such as gulls or cormorants [32]. Collected fish were placed in labeled polyethylene bags for storage at -20°C until processed when each was washed, weighed, and measured. Fish were identified to species when possible [33], otherwise to family. Since fish were in various stages of desiccation when collected, we dried fish at 50°C for 48-hrs then measured dry length and mass to develop comparable biomass estimates among individuals.

## Results and Discussion

### Monthly Water Quality

**Fig 2:**
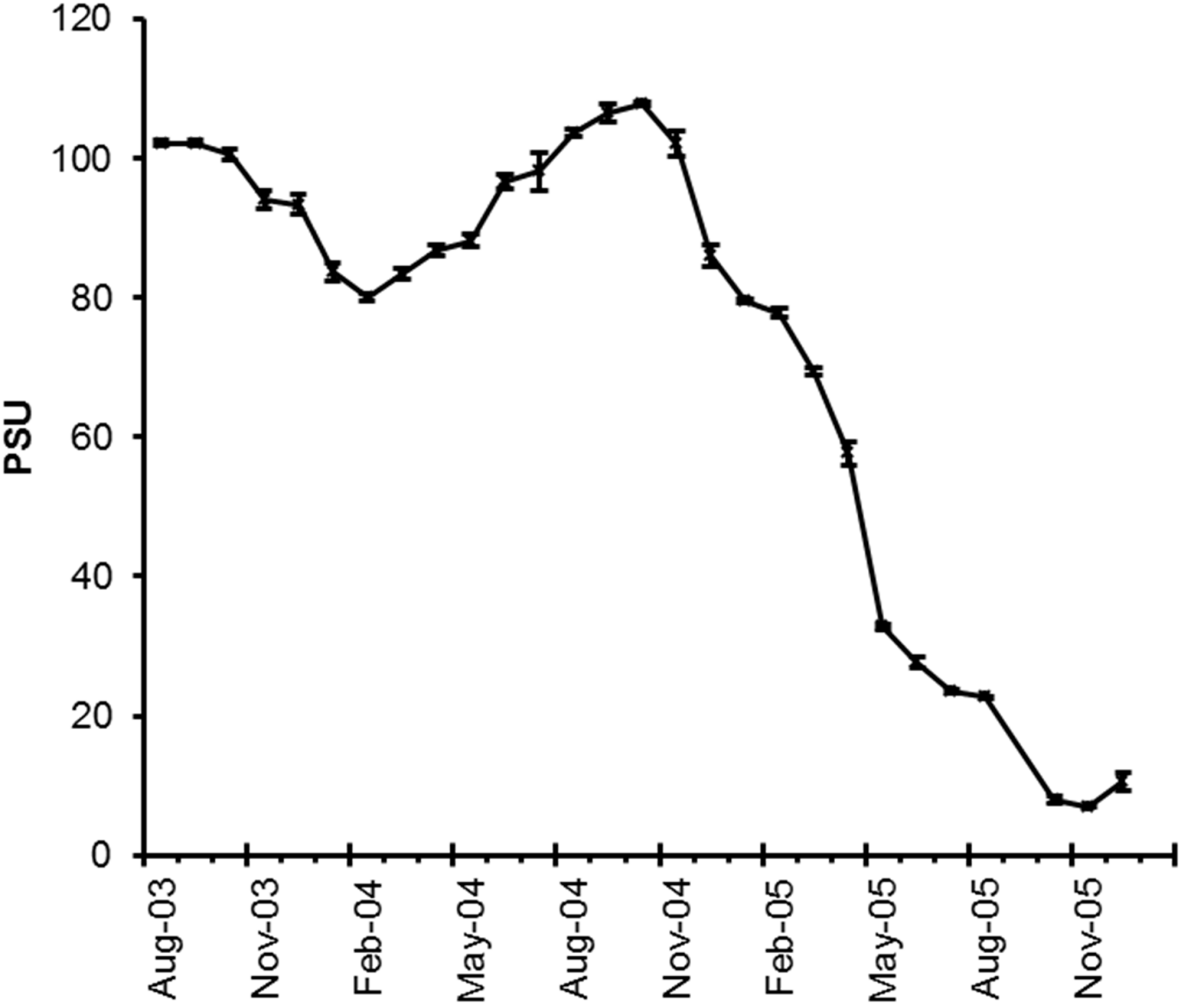
Monthly mean salinity (PSU; ± SE; *n* = 4) changes over the course of restoration in Pond A16. We used four measurements from around the pond to calculate SE where August 2005 corresponds with a period of hypoxia.

**Fig 3:**
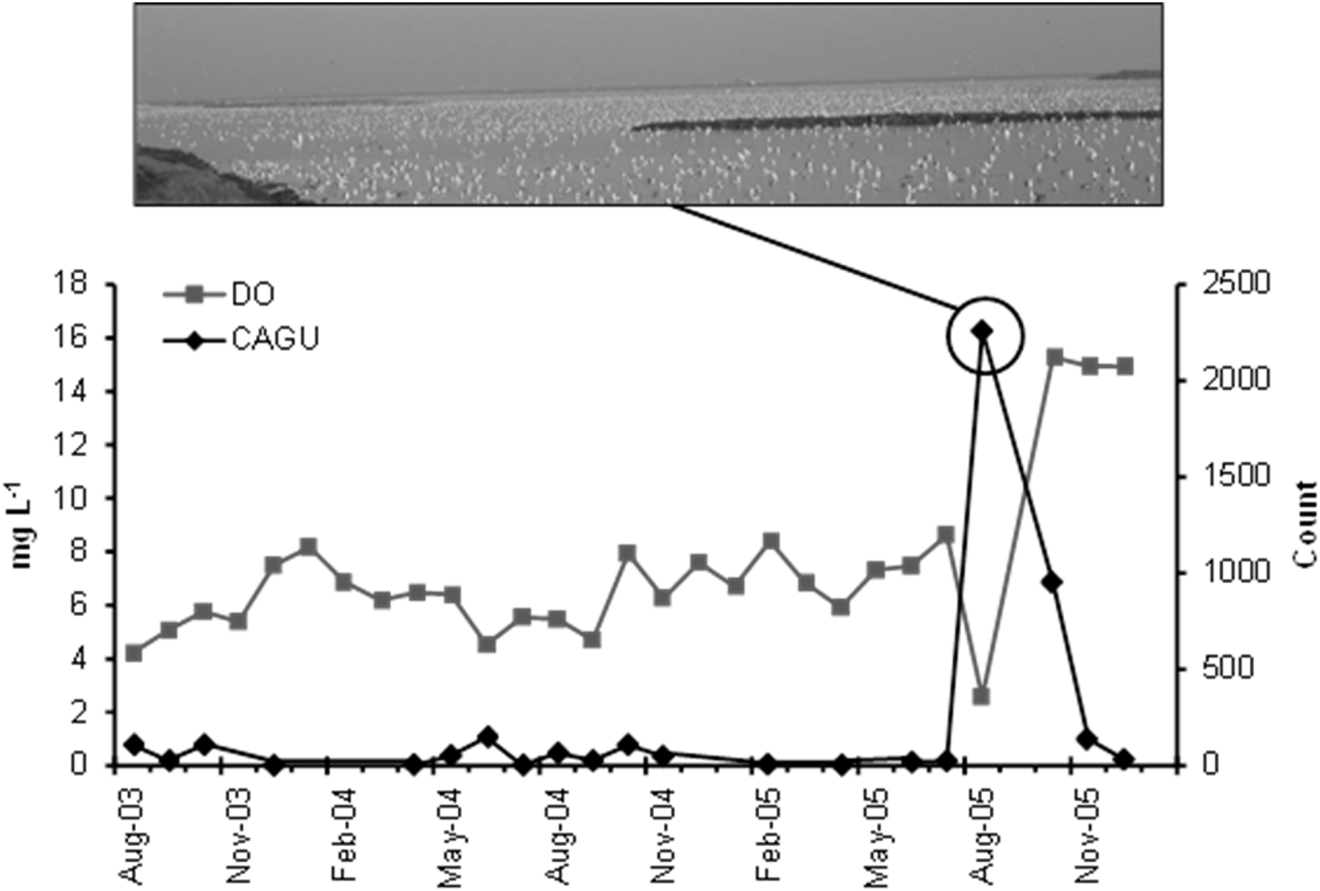
Monthly mean dissolved oxygen (DO; mg L^-1^; grey square line) from minisonde samples compared with monthly bird data for California Gulls (CAGU; count; black diamond line) from August 2003 through December 2005. The image is an example of CAGU on Pond A16 taken on 10 August 2005 which corresponded with a period of hypoxia.

We found strong seasonality and a dramatic decline in salinity over the study period (Fig 2). Prior to restoration actions, Pond A16 was hypersaline with greatest salinity in evaporative summer months (>100 PSU) and slightly lower salinity during winter with increased rainfall (> 80 PSU). Following initiation of circulation, salinity declined by an order of magnitude from the previous year to 8.0 PSU in the autumn. Unlike salinity, DO levels from monthly sampling remained stable and relatively high (> 4.2 mg L^-1^) throughout the study period except for the low DO event in August 2005 and the rapid DO increase later in the fall following this hypoxic event (Fig 3).

### Continuous Water Quality and Gate Management

**Fig 4:**
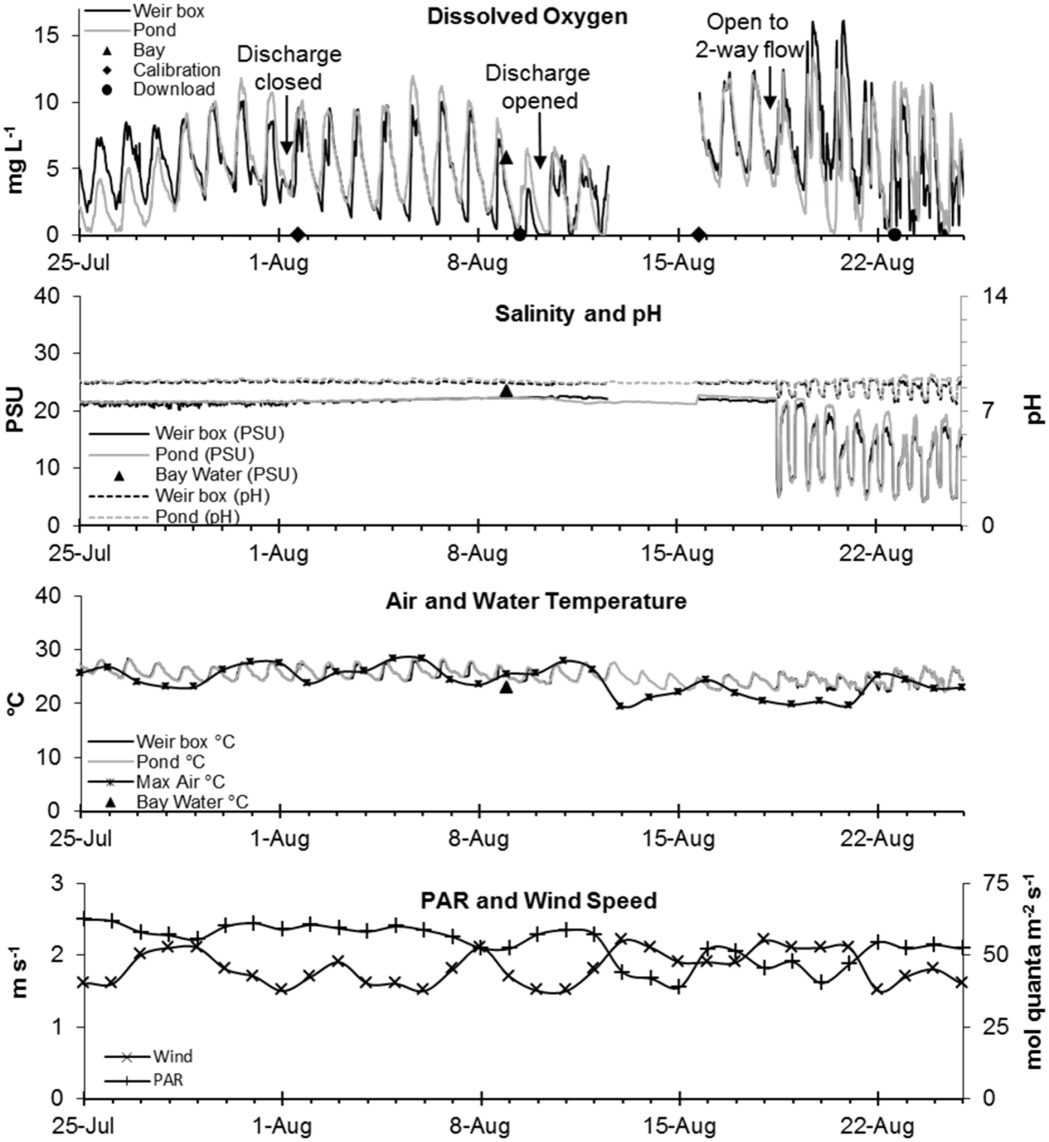
Fine scale water quality and environmental parameters of Pond A16 from 25 July through 25 August 2005. Water quality conditions from the pond (pale grey line) and weir box (dark grey) datasondes are depicted in the top three panels: Dissolved oxygen (DO; mg L^-1^), pH and salinity (PSU), water temperature (°C). Weather conditions are represent in the bottom two panels: air temperature (°C), photosynthetically active radiation (PAR; mol quanta m^-2^ s^-2^), and wind speed (m s^-1^). Data download (circles) and calibration (diamonds) occurred on alternating weeks. Triangles (top two panels) represent ambient South San Francisco Bay water conditions. The DO sensor of both datasondes were fouled and failed to log data from 12–15 August.

Continuous monitoring during August 2005 showed strong diurnal fluctuation in DO as well as periods of DO depletion interspersed with active gate management (Fig 4). After DO fell below a minimum threshold set by the Regional Water Quality Control Board (10^th^ percentile of daily DO measurements < 3.3 mg L^-1^), managers closed the discharge gate in Pond A16 on 1 August 2005 to protect receiving waters [34]. In the early mornings from 9–12 August 2005, DO dropped to 0.0 mg L^-1^ for both datasondes. During this period, algal mats and dead and dying fish were observed in great numbers within Pond A16. In an effort to save any remaining fish in Pond A16, managers responded by opening the discharge gates to allow one-way flow into Pond A16, but little immediate effect was recorded on DO concentration. Datasondes became fouled by algae from 12–15 August, but nocturnal minimum DO had rebounded by 15 August. On 18 August, the discharge gate was opened to normal two-way flow followed by increased fluctuation in salinity, pH, DO, and water temperature (Fig 4). Throughout the study period, weather remained relatively stable with the greatest fluctuation over 11–13 August when air temperature decreased 8°C, PAR decreased 14.7 mol quanta ^-2^ s^-1^, and wind speed increased 0.7 m s^-1^ (Fig 4).

### Prey Fish

**Fig 5:**
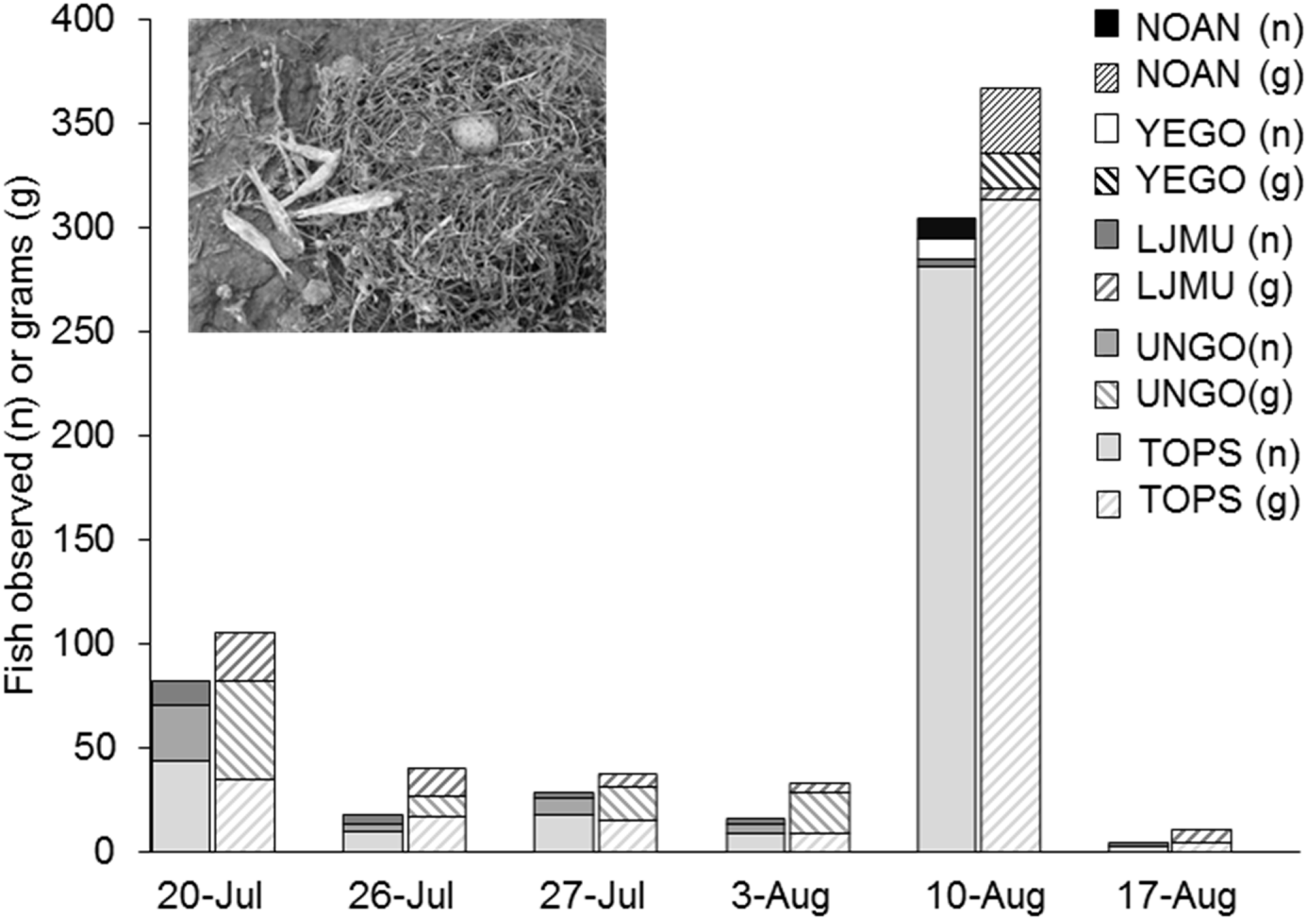
Count (n; solid colors) and dry weight (g; patterns) of fish collected at Forster’s Tern nesting colonies on Pond A16 from 20 July through 17 August 2005. The five taxa represented (Northern anchovy, NOAN; yellowfin goby, YEGO; longjaw mudsucker, LJMU; unknown goby, UNGO; topsmelt = TOPS), constituted 95% of count and dry weight observed.

During the period of time when chicks were on the islands (20 July to 17 August 2005), we collected 471 individual dropped fish of 9 species (primarily topsmelt) from the Forster’s Tern colony (Table 1). Numbers and biomass of dropped fish collected on the colony peaked from 4–10 August, corresponding to the same time as the depleted DO event. Greater than 66% of all dropped fish collected for the entire breeding season were collected during that week, which represented a ≥ 6-fold increase in the weekly fish biomass deposited at the islands by avian predators compared with the weeks prior and following the low-DO event. More than 98% of the dropped fish collected were topsmelt, longjaw mudsucker, yellowfin goby, Northern anchovy, and unidentified gobies (Fig 5). Northern anchovy, Mississippi silversides, and yellowfin goby were not observed on any other sampling date.

**Table 1:** Species and relative percent collected of 471 individual fish from the Forster’s Tern colony between 20 July and 17 August 2005.

Species	% of total
Topsmelt (*Atherinops affinis*)	76.4
Longjaw mudsucker (*Gillichthys mirabilis*)	5.4
Yellowfin goby (*Acanthogobius flavimanus*)	2.1
Northern anchovy (*Engraulis mordax*)	2.1
Shiner surfperch (*Cymatogaster aggregata*)	1.7
Mississippi silverside (*Menidia audens*)	0.6
Bluegill (*Lepomis macrochirus*)	0.2
Largemouth bass (*Micropterus salmoides*)	0.2
Starry flounder (*Platichthys stellatus*)	0.2
Unidentified Goby sp. (*Acanthogobius spp*.*)*	9.3
Unknown	1.6

### Gulls and Terns

California Gull abundance in Pond A16 was low from August 2003 through July 2005 (Fig 3). A dramatic shift occurred when California Gulls increased by two orders of magnitude from a monthly mean of 23 individuals in July 2005 (n = 16), to 2,258 in August 2005. Using photographic survey methodology, 2,030 California Gulls were counted on 10 August 2005 directly corresponding to the lowest observed DO.

We captured 254 Forster’s Tern chicks and recaptured 359 banded chicks during the nesting season at Pond A16. Survival models that included both calendar date and gull response were strongly supported (Σw_i_ = 0.821, Table 2). Forster’s Tern chick survival increased as the breeding season progressed, but gull response had a strong negative effect on chick survival. Encounter probabilities were best described by a model containing calendar date and chick age (as well as by the categorical factor that accounted for fledging age), however a similar model that did not include chick age was supported nearly as well (ΔQAIC_c_ = 0.088).

**Table 2:** Model selection table for Forster’s Tern chick daily survival rates.

Apparent Survival Probability (Phi)	Capture Probability (P)	k	Deviance	QAICc	ΔQAICc	Akaike weight	Evidence Ratio
gull.invasion + time	fledged + chick.age + time	7	851.65	865.83	0.00	0.37	1.00
gull.invasion + time	fledged + time	6	853.78	865.92	0.09	0.35	1.05
gull.invasion + time	fledged + chick.age	6	857.16	869.30	3.46	0.06	5.65
gull.invasion	fledged + time	5	860.00	870.09	4.26	0.04	8.42
gull.invasion + time	fledged	5	860.31	870.41	4.58	0.04	9.86
gull.invasion	fledged + chick.age + time	6	858.57	870.71	4.88	0.03	11.46
gull.invasion + chick.age	fledged + chick.age + time	7	856.79	870.98	5.14	0.03	13.08
gull.invasion + chick.age	fledged + time	6	859.87	872.01	6.17	0.02	21.88
chick.age + time	fledged + chick.age	6	860.56	872.70	6.86	0.01	30.90
intercept only	fledged + time	4	865.61	873.68	7.85	0.01	50.55
intercept only	fledged + chick.age + time	5	863.69	873.79	7.96	0.01	53.44
chick.age	fledged + chick.age + time	6	861.83	873.96	8.13	0.01	58.28
chick.age + time	fledged + chick.age + time	7	859.81	873.99	8.16	0.01	59.15
time	fledged + chick.age + time	6	862.05	874.19	8.35	0.01	65.16
time	fledged + chick.age	5	864.53	874.63	8.79	0.00	81.07
time	fledged + time	5	864.82	874.92	9.09	0.00	94.14
chick.age	fledged + time	5	865.04	875.14	9.30	0.00	104.75
chick.age + time	fledged + time	6	864.43	876.57	10.73	0.00	213.91
time	fledged	4	872.05	880.11	14.28	0.00	1261.50
chick.age + time	fledged	5	871.70	881.80	15.96	0.00	2927.06
chick.age	fledged + chick.age	5	873.22	883.32	17.48	0.00	6260.74
gull.invasion + chick.age	fledged + chick.age	6	872.55	884.69	18.86	0.00	12453.72
intercept only	fledged + chick.age	4	878.46	886.53	20.69	0.00	31161.07
gull.invasion	fledged + chick.age	5	877.32	887.42	21.59	0.00	48681.93
gull.invasion	fledged	4	880.65	888.72	22.88	0.00	93162.88
intercept only	fledged	3	883.03	889.07	23.23	0.00	110999.58
gull.invasion + chick.age	fledged	5	880.27	890.37	24.54	0.00	212853.23
chick.age	fledged	4	882.32	890.38	24.55	0.00	214317.72

**Fig 6:**
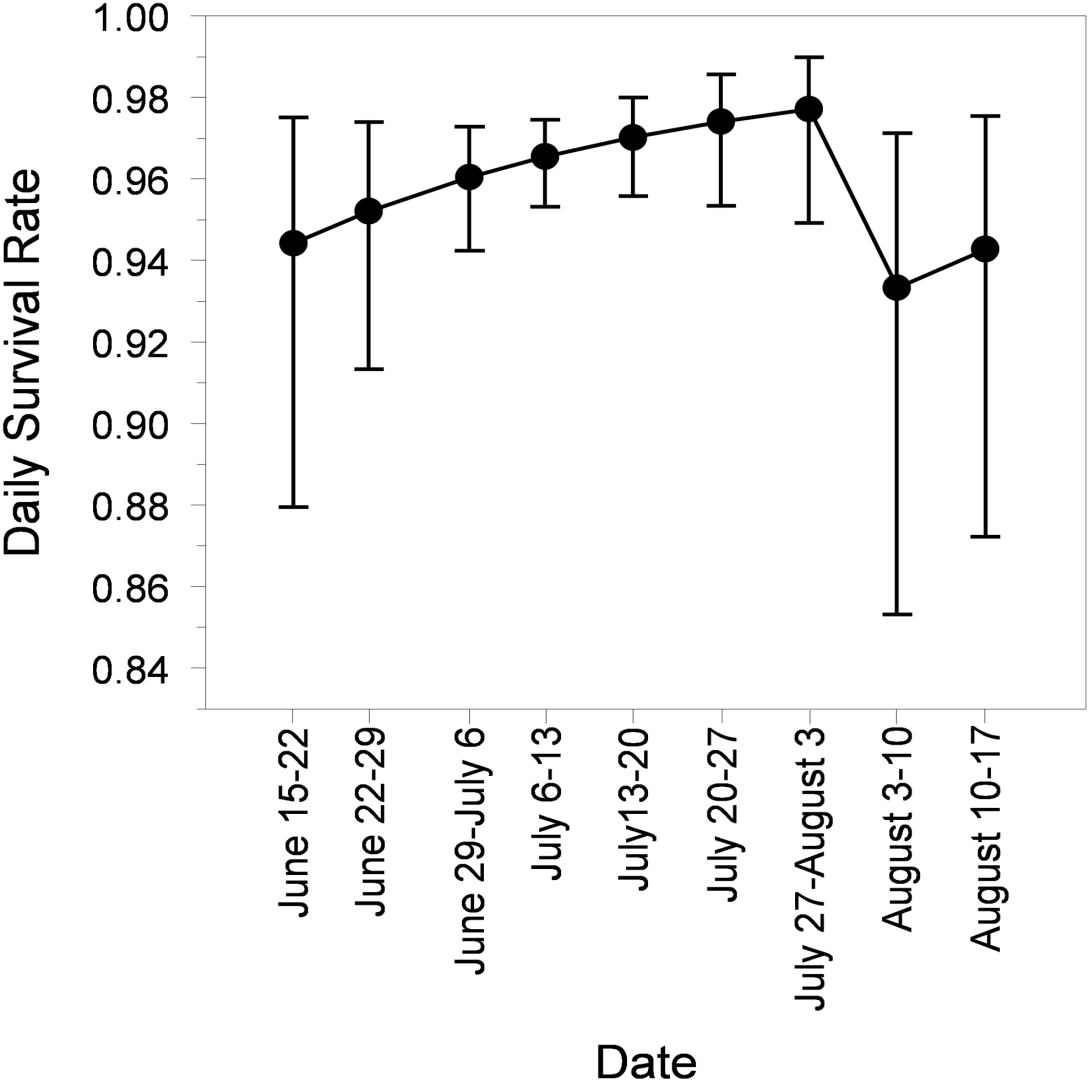
Daily survival rate with 95% Confidence Intervals (CI) of Forster’s Tern chicks during each of nine intervals between 10 capture events at Pond A16 in San Francisco Bay during the breeding season. California Gulls started using Pond A16 heavily on 3 August 2005, and Forster’s Tern chick survival declined significantly thereafter.

Daily survival rates declined from 0.977 ± 0.009 (mean ± SE) the week prior to the gull response to 0.933 ± 0.028 (-4.5%) and 0.943 ± 0.024 (-3.5%) for the two periods (3–10 and 10–17 August 2005) after gull response, respectively, based on model-averaged estimates (Fig 6). The decline of Forster’s Tern chick daily survival from 0.977 to 0.933 after the gull response would correspond to a 72% decline in chick survival over the typical 28-day period from hatching to fledging [19]. Gull response had very high relative importance (Σw_i_ = 0.941) in our models describing chick survival. Additionally, the evidence ratio for the most supported models with and without the gull response (gull response + calendar date; *w*_*i*_ = 0.367; chick age + calendar date; *w*_*i*_ = 0.0119) indicates that our most parsimonious model with gull response was 31-times more likely than the best model without the gull response. When compared to a model with only calendar date (*w*_*i*_ = 0.00564), the model that included the gull response event was 65-times more likely. During the entire nesting season, we collected 28 Forster’s Tern chicks that were found dead within Pond A16 during weekly visits. Only three of these dead chicks were found during the period of gull invasion, indicating that most of the chick mortalities during the gull invasion were caused by predation and not exposure. Elsewhere we have shown that many predation events result in chicks being depredated and carried off of nesting islands and back to gull nesting colonies [35, 36].

## Conclusions

**Fig 7:**
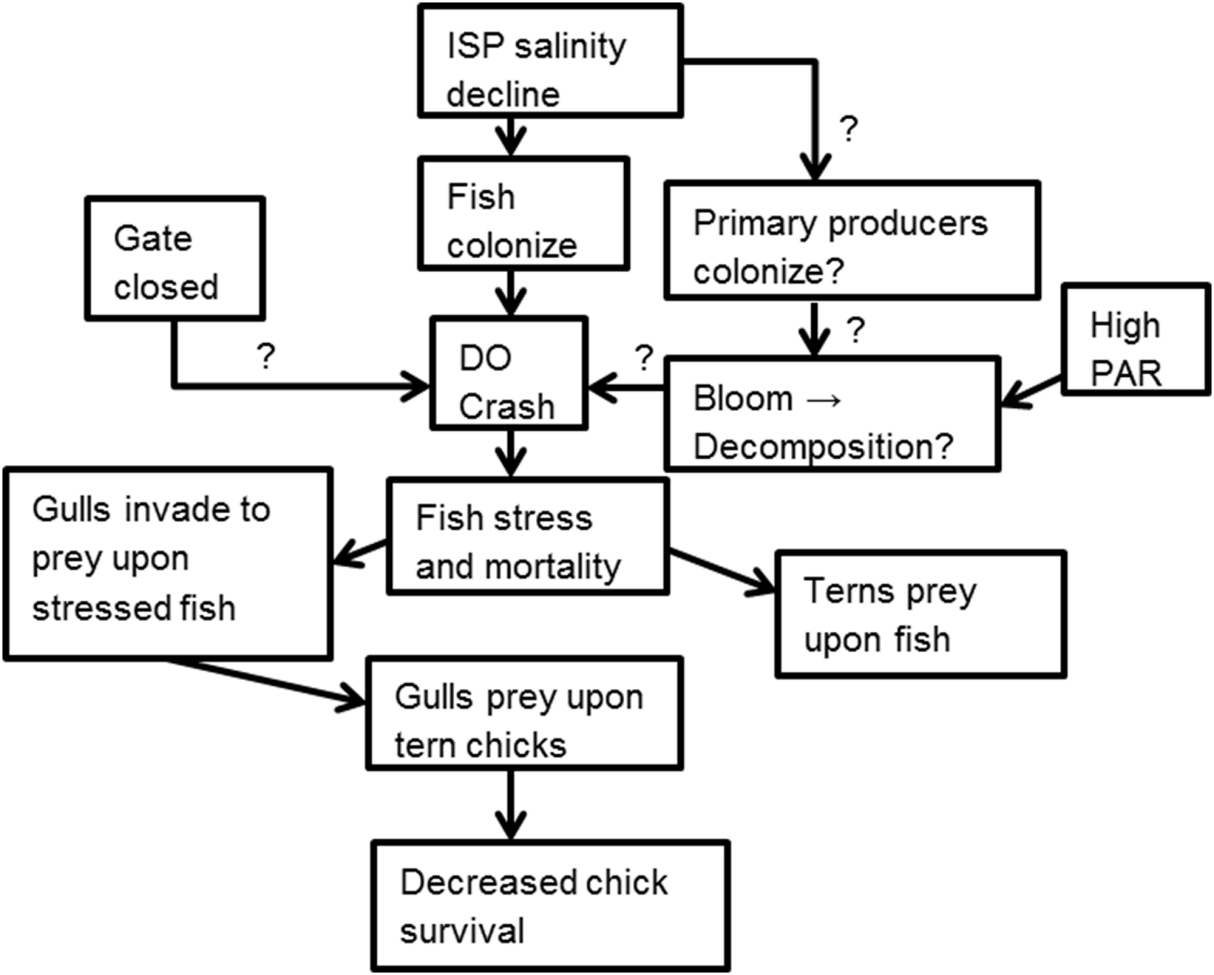
Chain of events and trophic interactions of Pond A16 concomitant with the low DO and gull intrusion events. Salinity reduction resulted from management actions during the Initial Stewardship Plan (ISP) of the South Bay Salt Pond Restoration Project in San Francisco Bay.

Prior to lower-salinity bay water entering the former salt ponds, hyperhaline salt production ponds in San Francisco Bay maintained halophytic algae, bacteria, and arthropods but exceeded tolerance thresholds for most fish [17, 22]. Salinity reduction supported broader colonization of fish species, including topsmelt and longjaw mudsucker below 80 PSU, yellowfin goby below 62 PSU, and Northern anchovy below 30 PSU [22, 37, 38]. Increased prey resources from newly-colonized fish species likely enhanced foraging habitat for piscivorous birds [22, 38]. However, management at Pond A16 was perturbed by an unexpected cascade of biochemical and trophic interactions (Fig 7).

Following salinity reduction that enabled broader fish colonization, eutrophication of the pond compounded with closure of the gates created an ecological trap (*sensu*, [39]) with hypoxic conditions followed by a fish kill. During that period, salinity, pH, and temperature remained stable, but the 10^th^ percentile of DO fell below 1.0 mg L^-1^ for ≥4 consecutive days. Minimum DO tolerance was exceeded for yellowfin goby (6.8 mg/L), topsmelt (2.5 mg/L), longjaw mudsucker (1.6 mg/L), and Northern anchovy (1.0 mg/L; [38, 40, 41]). Vulnerability of these fish species to predation likely increased with the onset of surface respiration [12, 13, 34].

Piscivorous birds can exhibit behavioral plasticity to benefit from aerobically-stressed fish. Little Egrets (*Egretta garzetta*) in salt marshes shifted from solitary to aggregated foraging and foraging success increased by 3-fold during predictable, early-morning hypoxia [14]. Likewise, we observed ≥6-fold increase in the number and biomass of fish deposited at the Forster’s Tern colony during hypoxic conditions. However, despite increased access to prey fish and wide-ranging foraging abilities of parents [42], Forster’s Tern chick daily survival rates declined from 0.977 to 0.933 after the gull response which corresponds to a 72% reduction in chick survival over the typical 28-day period from hatching to fledging [19]. Forster’s Tern chicks already have relatively low survival rates (22%) primarily due to predation by California Gulls [40]. In fact, California Gulls caused 54% of Forster’s Tern and 55% of American Avocet chick deaths in related studies in San Francisco Bay, California [35, 36], before they reach postfledging age where juvenile tern survival rates are considerably higher [43]. Thus, tern chick survival rates appear to have been reduced even more than their normally low levels due to the unanticipated behavioral response of California Gulls being attracted to the site by the high abundance of dying, aerobically-stressed fish. The Forster’s Tern chicks became an additional prey source for the over 2,000 gulls, dramatically reducing Forster’s tern chick survival.

The use of dropped fish as an index of increased foraging activity is not ideal. Increased foraging intensity when fish were more abundant could result in an increase in dropped fish. In that scenario, the number of fish dropped at when fish density is greater might result in an overestimate of fish abundance relative to low abundance conditions. However, we are simply using dropped fish as an index of a fish kill which occurred on the pond since we had no other data on fish abundance. The detrimental effects of low DO on aquatic organisms are well described [11, 13]; however, this is the only study we are aware of that measured the unintended consequences of changing abiotic conditions to manage a saline pond restoration where shifts in DO concentrations altered species interactions [10, 16]. In Chesapeake Bay, varying DO have had widely different effects on predator-prey interactions depending on responses of individual species [10]. However, our case study indicates that these effects may cascade with long-term population-level impacts to upper trophic levels that otherwise would be expected to be immune to anoxic conditions.

Our results illustrate the importance of considering multiple aspects of water quality and species interactions when undertaking restoration efforts. Furthermore, it highlights the value of using adaptive restoration [2] and regular monitoring to better understand complex system responses to management actions. It shows the need to balance the sometimes competing management needs of reducing salinity levels, protecting receiving waters, and maintaining adequate dissolved oxygen levels suitable for supporting aquatic fauna.
